# Sequential therapy with anlotinib and radioiodine for distant metastatic differentiated thyroid cancer: a phase II clinical trial

**DOI:** 10.1093/oncolo/oyag426

**Published:** 2026-10-22

**Authors:** Hongjun Song, Chentian Shen, Youben Fan, Zhiyan Liu, Yonggang Wang, Quanyong Luo, Libo Chen

**Affiliations:** Department of Nuclear Medicine, Shanghai Sixth People’s Hospital, Affiliated to Shanghai Jiao Tong University School of Medicine, 600 Yishan Road, Shanghai 200233, China; Department of Nuclear Medicine, Shanghai Sixth People’s Hospital, Affiliated to Shanghai Jiao Tong University School of Medicine, 600 Yishan Road, Shanghai 200233, China; Department of Surgery, Shanghai Sixth People’s Hospital, Affiliated to Shanghai Jiao Tong University School of Medicine, 600 Yishan Road, Shanghai 200233, China; Department of Pathology, Shanghai Sixth People’s Hospital, Affiliated to Shanghai Jiao Tong University School of Medicine, 600 Yishan Road, Shanghai 200233, China; Department of Oncology, Shanghai Sixth People’s Hospital, Affiliated to Shanghai Jiao Tong University School of Medicine, 600 Yishan Road, Shanghai 200233, China; Department of Nuclear Medicine, Shanghai Sixth People’s Hospital, Affiliated to Shanghai Jiao Tong University School of Medicine, 600 Yishan Road, Shanghai 200233, China; Department of Nuclear Medicine, Shanghai Sixth People’s Hospital, Affiliated to Shanghai Jiao Tong University School of Medicine, 600 Yishan Road, Shanghai 200233, China

**Keywords:** differentiated thyroid carcinoma, metastasis, radioiodine, tyrosine kinase inhibitor, anlotinib

## Abstract

**Background:**

Radioiodine (RAI) alone exhibited limited efficacy in distant metastatic differentiated thyroid cancer (DTC). We hypothesized that pretreatment with anlotinib, a potent antiangiogenic tyrosine kinase inhibitor, would reduce tumor burden and improve therapeutic response.

**Methods:**

This prospective, single-arm phase II trial enrolled patients with measurable distant metastatic DTC. All patients received anlotinib 12 mg/day orally for 12 weeks. After confirmation of RAI-avid target lesions on diagnostic ^1^^3^^1^I-WBS, patients were treated with 7.4 GBq RAI and followed up for another 12 weeks. Sequential therapy continued until no effect of RAI could be anticipated. Primary endpoints were objective response rate (ORR) and safety. Registered with the Chinese Clinical Trial Registry (ChiCTR2100045178; https://www.chictr.org.cn).

**Results:**

24 patients were enrolled. 14 (58%) were excluded due to non-RAI-avid target lesions; 10 (42%) with RAI-avid lesions received sequential therapy (median, 4.5 cycles). ORR was 60% (all partial responses), disease control rate 100%. With median follow-up 34.5 months, no disease progression or death occurred. In the subgroup of 8 patients with bone target lesions, ORR was 50%. Grade 3 anlotinib-related adverse events occurred in 30% of patients; no grade 4/5 toxicities or grade ≥2 RAI-related events were observed.

**Conclusion:**

Sequential anlotinib followed by RAI therapy demonstrated promising antitumor activity (ORR 60%) and favorable safety in RAI-avid distant metastatic DTC, particularly in bone metastases. These findings are hypothesis-generating and require validation in a randomized trial with an RAI monotherapy control arm.

Lessons learned

- In 10 evaluable patients with RAI-avid distant metastatic DTC, a 12-week anlotinib prime followed by 7.4 GBq RAI achieved an ORR of 60% (6/10 PR), a DCR of 100%, and a 50% ORR in 8 patients with bone lesions. Safety was favorable: grade 3 anlotinib-related AEs occurred in 30%, with no grade 4/5 toxicities and no grade ≥2 RAI-related AEs. At median follow-up of 34.5 months, no disease progression or death occurred, and median PFS was not reached.
- The RAI-avidity assessment stage had a high withdrawal rate (14/24, 58%), reducing the evaluable population to 10 patients; the trial therefore did not reach its planned 19 evaluable patients. Future sequential TKI–RAI trials should incorporate pre-enrollment screening for RAI-avid target lesions to optimize patient selection and ensure adequate accrual of evaluable patients.
- The small evaluable sample limited statistical power, and the absence of an RAI monotherapy control arm precludes causal attribution of responses to the sequential regimen. Although the preliminary efficacy and safety signals were encouraging, they cannot establish superiority over RAI alone and should be considered hypothesis-generating. Future studies should enroll more patients and include an RAI monotherapy arm in a rigorous randomized design.

## Trial information

**Table:** 

**TRIAL INFORMATION**	
**Disease**	Distant metastatic differentiated thyroid cancer with RAI-avid target lesions
**Stage of disease**	Metastatic (predominantly stage IV)
**Prior therapy**	Total or near-total thyroidectomy; no prior VEGFR-TKI exposure
**Type of study**	Prospective, open-label, single-arm phase II study
**Primary endpoints**	ORR by RECIST v1.1 and safety (TEAEs per CTCAE v5.0)
**Secondary endpoints**	Biochemical remission rate, time-to-response, and progression-free survival
**Additional details of endpoints or study design** Fleming single-stage design assumed an ORR of 10% with RAI alone and targeted 50% with sequential therapy (one-sided α = 0.05; power = 80%; planned 19 evaluable patients). Twenty-four patients were enrolled; 14 (58%) were excluded for non-RAI-avid target lesions on diagnostic ^1^^3^^1^I whole-body scan; 10 (42%) received RAI and were evaluable. Study Design (shown in Figure 1): Patients received oral anlotinib at an initial dose of 12 mg once daily (2 weeks on/1 week off) for 12 weeks, with levothyroxine withdrawal during weeks 9-12. Patients with RAI-avid target lesions on diagnostic ^131^I whole-body scan^1^ then received 7.4 GBq (200 mCi) RAI and were followed for another 12 weeks; those with non-RAI-avid lesions were withdrawn and managed per off-study protocol.^2–5^ Cycles repeated until complete response, progression, non-RAI-avidity, no biochemical response, or intolerable toxicity.	

## Drug information

**Table:** 

**DRUG INFORMATION**	
**Generic/working name**	Anlotinib
**Company name**	Chia-tai Tianqing Pharmaceutical Co., Ltd.
**Drug type**	Small-molecule
**Drug class**	Multi-target tyrosine kinase inhibitor (VEGFR, FGFR, PDGFR)
**Dose**	12 mg once daily; dose modifications permitted according to observed toxicities
**Route**	Oral
**Schedule of administration**	Once daily, 2 weeks on/1 week off, for 12 weeks

**Table:** 

**DRUG INFORMATION**	
**Generic/working name**	Radioiodine (^1^^3^^1^I)
**Drug type**	Radioactive isotope
**Drug class**	Radioiodine
**Dose**	7.4 GBq (200 mCi)
**Route**	Oral
**Schedule of administration**	Single dose per cycle after diagnostic ^1^^3^^1^I whole-body scan confirmed RAI-avid target lesions.

## Patient characteristics

From June 26, 2021, to October 21, 2024, 24 patients with distant metastatic DTC were enrolled. By the data cutoff (July 30, 2025), 14 (58%) were excluded for non-RAI-avid target lesions on diagnostic ^1^^3^^1^I whole-body scan (Dx-WBS), leaving 10 (42%) who received RAI and completed the entire sequential therapy. These 10 patients were considered evaluable and analyzed for efficacy and safety. Baseline characteristics are shown in Table 1. Complete eligibility criteria are provided in the Supplement.

**Table 1.** Baseline characteristics of patients eligible for data analysis (*n* = 10).

No.	Sex	Age (years)	Histology	Primary tumor size (mm)	Distribution of metastases	Location of target lesion (s)	Sum of target lesion sizes (mm)	Tg (ng/mL)	TgAb (IU/mL)	ECOG-PS score	Cycle (s)
**02**	Female	67	FTC	22	L, B (Multiple)	B	125	25 000	48.3	1	4
**04**	Male	60	PTC	1.5	L, B (Solitary), Mu	L	52	303	13.5	0	7
**12**	Female	69	OCA	1.5	L, B (Solitary)	B	42	434	11.2	1	5
**13**	Female	66	FTC	21	L, B (Multiple)	B	64	10 997	19.5	1	5
**14**	Male	61	FTC	25	L, B (Solitary)	B	96	25 000	18.5	1	2
**16**	Male	67	FTC	54	Mediastinal LN, L	Mediastinal LN	50	694	21.7	1	5
**17**	Female	61	FTC	12	L, B (Multiple)	B	76	20 281	77.4	1	5
**21**	Male	66	FTC	25	L, B (Multiple)	B	54	20 000	22.9	1	3
**23**	Male	45	FTC	28	L, B (Multiple)	L, B	84	21 216	85.5	1	3
**25**	Male	63	FTC	20	L, B (Multiple)	B	144	7536	33.6	1	2

Abbreviations: B, bone; FTC, follicular thyroid cancer; L, lung; LN, lymph node; Mu, muscle; OCA, Oncocytic carcinoma of the thyroid; PTC, papillary thyroid cancer; Cycle (s) means numbers of sequential anlotinib-131I therapy cycle.

## Primary assessment method

**Table:** 

**PRIMARY ASSESSMENT METHOD**	
**Title**	Objective response rate and safety
**Number of patients screened**	24
**Number of patients enrolled**	24
**Number of patients evaluable for toxicity**	10
**Number of patients evaluated for efficacy**	10
**Evaluation method**	Tumor response was assessed by investigators by CT per RECIST v1.1, RAI avidity by diagnostic ^1^^3^^1^I whole-body scan, and AEs per CTCAE v5.0. Secondary endpoints included biochemical remission rate, structural TTR (from anlotinib initiation to first remission), biochemical TTR (shortest time to biochemical remission by δTg_on_% criteria), and PFS (from anlotinib initiation to progression or death). Biochemical response was assessed using suppressed Tg_on_ levothyroxine (Tg_on_): δTg_on_% = [(posttreatment Tg_on_ − baseline Tg_on_)/baseline Tg_on_] × 100%. Patients were classified as biochemical remission (δTg_on_% < −25.0%), biochemically stable disease (−25.0% ≤ δTg_on_% ≤ 25.0%), or biochemically progressive disease (δTg_on_% > 25.0%). Blood pressure was recorded daily; medical history and physical examination were performed every 4 weeks after anlotinib initiation. Blood samples were collected for CBC, metabolic panel, TSH, Tg, and TgAb. Tumor response was assessed at the end of weeks 8, 12, and 24 of each cycle. AEs were graded per CTCAE v5.0 at each visit. Details are provided in Figure 1.

## Outcome notes

Of 24 enrolled patients, 14 (58%) were excluded because target lesions were non-RAI-avid on diagnostic ^1^^3^^1^I whole-body scan; 10 (42%) with RAI-avid lesions received sequential therapy (with a median cycle of 4.5, range, 2-7 cycles)) and were evaluable (Figure 2A, swimmer plot). Eight (80%) of 10 patients were still receiving treatment at the date of data cutoff (Figure 2A). Two patients discontinued treatment due to financial reason (*n* = 1) or voluntary option for surgical resection of the target lesion (*n* = 1).

**Figure 1.**
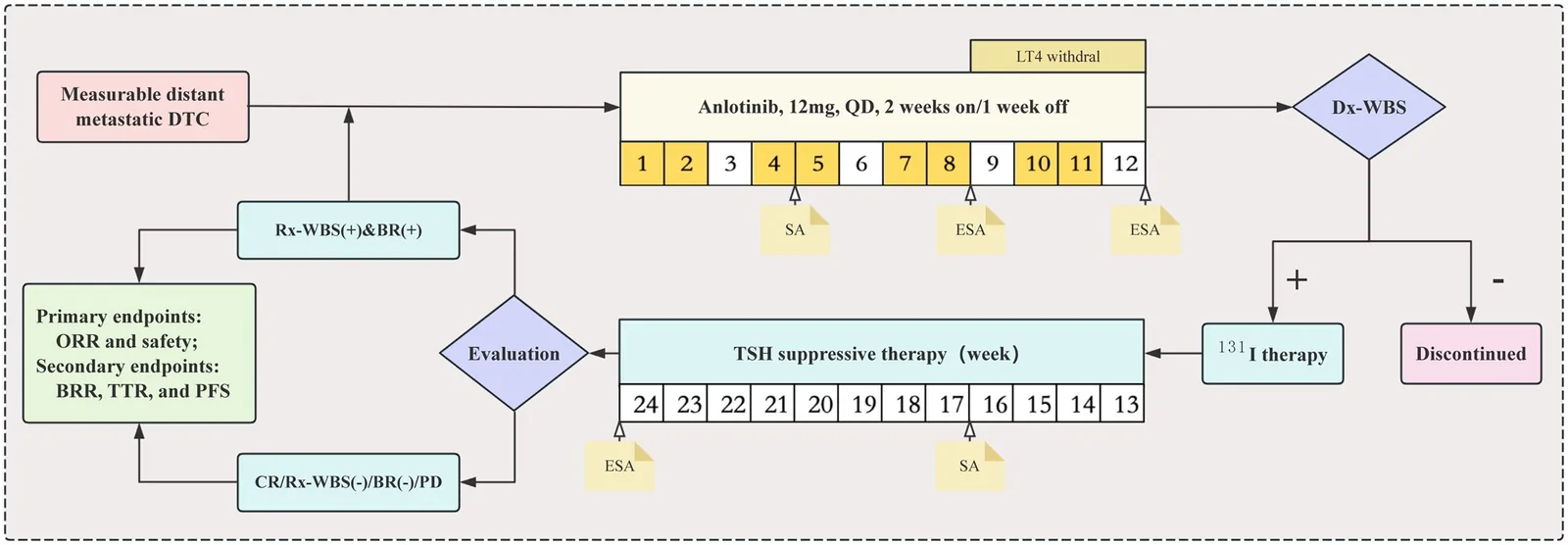
The study design of sequential therapy of anlotinib and RAI. Flow diagram showing 12-week anlotinib priming, diagnostic ^1^^3^^1^I whole-body scan, RAI 7.4 GBq treatment, 12-week follow-up, and continuation criteria. BR, biochemical remission; CR, complete response; DTC, differentiated thyroid cancer; Dx-WBS, diagnostic ^1^^3^^1^I whole-body scan; ESA, efficacy and safety assessments; ORR, objective response rate; PD, progressive disease; PFS, progression-free survival; Rx-WBS, post-therapeutic whole-body scan; SA, safety assessments; TTR, time-to-response.

**Figure 2.**
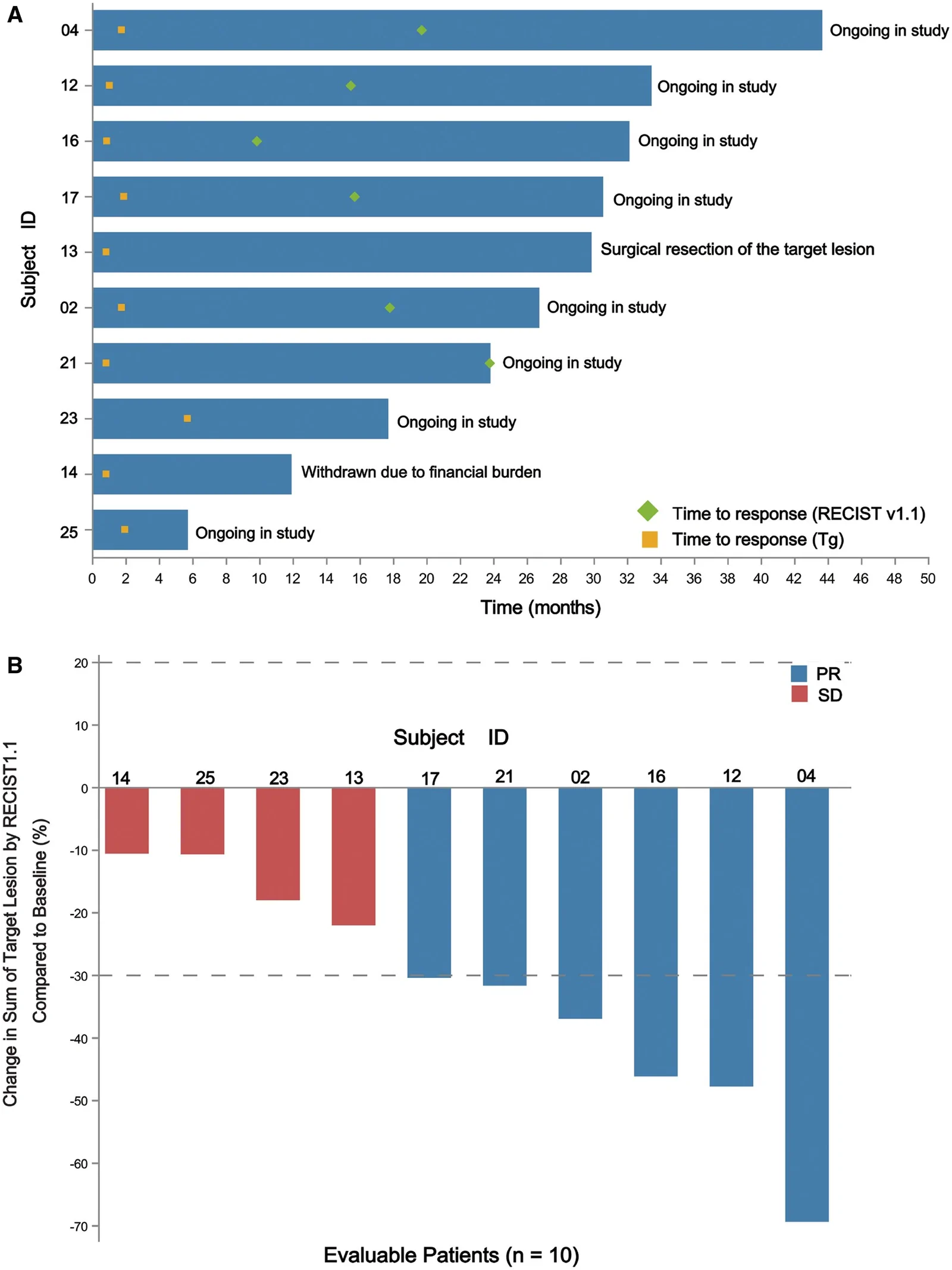
(A) Swimmer’s plot of 10 patients’ treatment study duration and time-to-response. Swimmer plot showing individual treatment courses for 10 patients, with biochemical and structural time-to-response markers. Each bar represents a patient. Text on right side of each bar represents reason for off study. The yellow squares represent biochemical TTR; the green diamonds represent structural TTR. (B) Best overall percentage of change from baseline in target lesion measurement. Waterfall plot showing best percentage change in target-lesion size for each evaluable patient, categorized as partial response or stable disease. Baseline radiographic measurements of target lesions were compared with measurements over the course of the study to determine the best change in target lesion size for each patient with data. PR, partial response; RECIST, Response Evaluation Criteria in Solid Tumors; SD, stable disease; TTR, time-to-response.

Throughout the study, no patients achieved CR and 6 patients (60%) attained a confirmed partial response (PR), yielding an ORR of 60% (95% CI, 19%-81%) per RECIST v1.1 (Figure 2B). The stable disease (SD) was observed in 4 patients (40%). The median sum of longest diameter of target lesions decreased from 70.0 mm (IQR, 51.5-100.5 mm) at baseline to 51.5 mm (IQR, 25.8-80.8 mm) post-treatment (*P* < .001), with a mean reduction in lesion diameter (mean ± standard deviation) of 21% ± 12%. Substantial tumor shrinkage was observed, with representative cases showing the reduction of lung and bone lesions in Figure 3. The median structural TTR was 15.7 months (95% CI, 11.1 to 20.3 months; Figure 2A). No disease progression or death occurred with a median follow-up time of 34.5 months (95% CI, 21-44 months). The median PFS data has not yet been reached. In the subgroup of 8 patients with bone target lesions, 4 (50%) achieved PR and 4 (50%) were assessed with SD, corresponding to an ORR of 50% (Figure 4).

**Figure 3.**
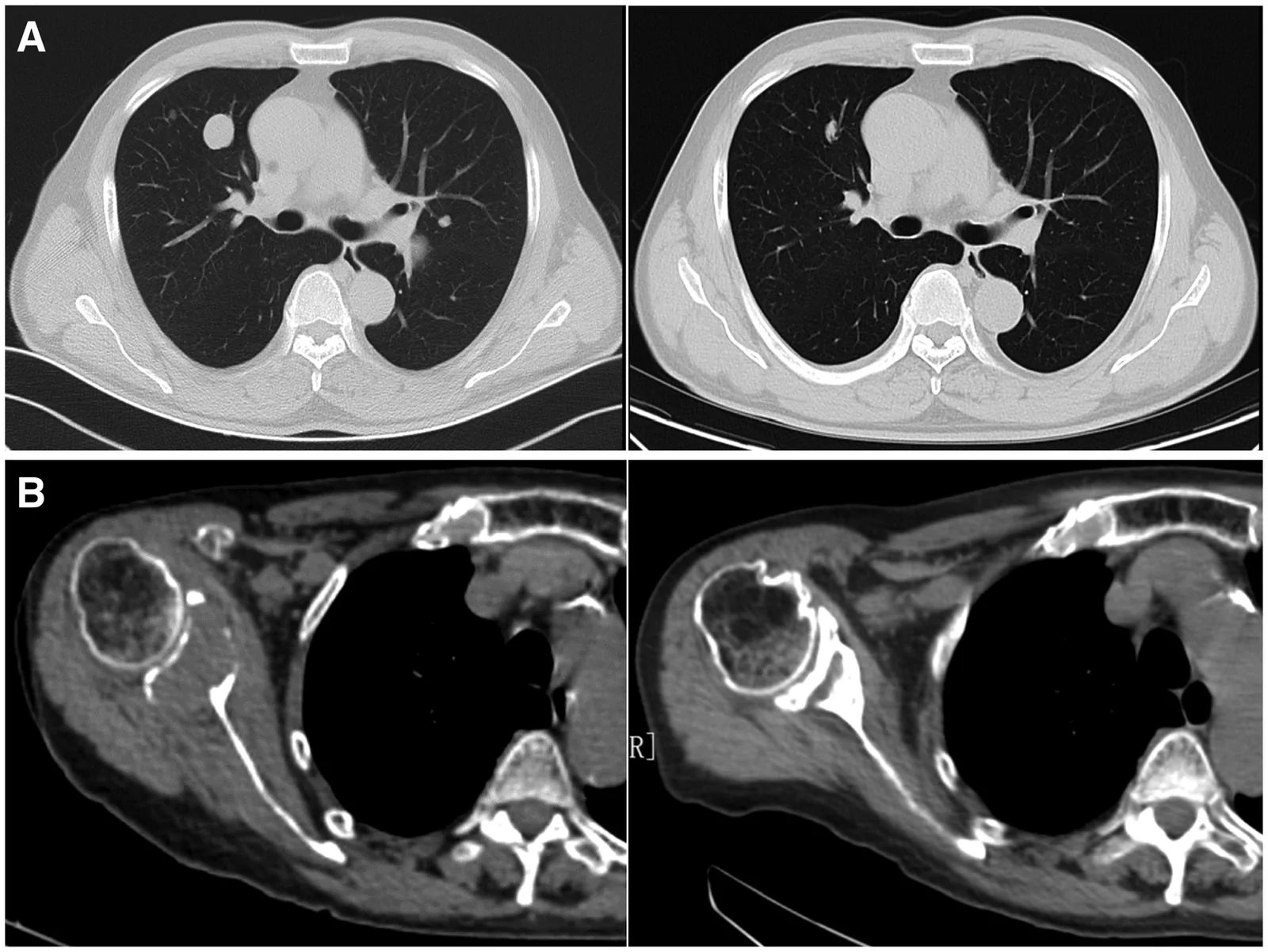
Representative CT responses. CT images from 2 patients showing reduction in lung and bone target lesions after sequential anlotinib and ^1^^3^^1^I therapy. (A) A 60-year-old man with papillary thyroid cancer had metastatic disease in the lung (upper). Computed tomography (CT) scans confirm partial response in target lesions (lower) after 3 cycles of sequential therapy of anlotinib and ^131^I. (B) A 69-year-old woman with oncocytic carcinoma of the thyroid had lung and right scapula metastases (upper). CT scans show marked improvement in the burden of right scapula disease after 4 cycles of sequential therapy of anlotinib and ^131^I (lower).

**Figure 4.**
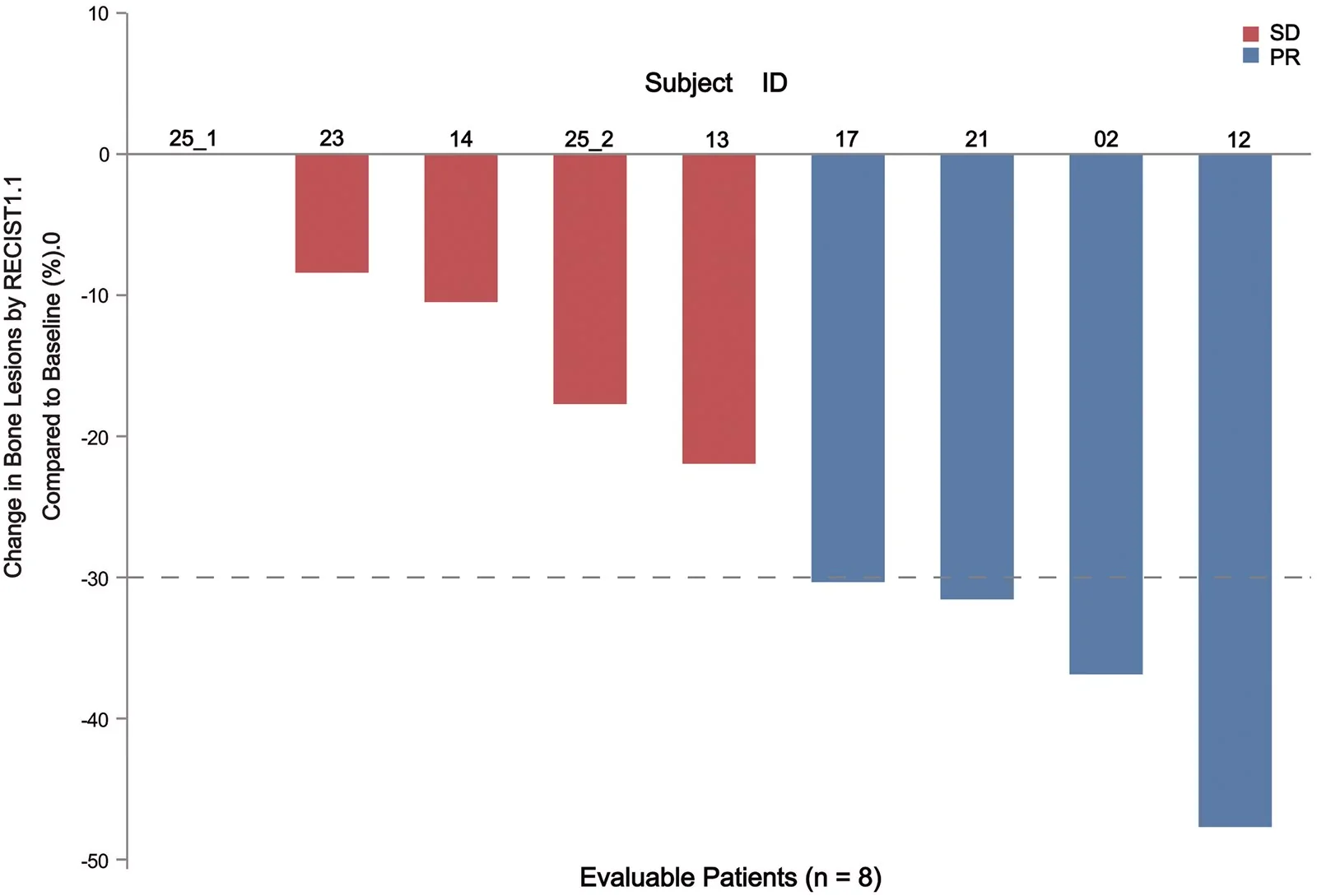
Best overall percentage of change from baseline in target lesion in bones. Baseline radiographic measurements of target lesions were compared with measurements over the course of the study to determine the best change in target lesion size for each patient with data. Each bar represents 1 bone target lesion. Patient 25 had 2 separate bone target lesions, shown as 25_1 and 25_2; therefore, the 9 bars correspond to 8 unique patients (*n* = 8) with a total of 9 evaluable bone target lesions. The ORR in the bone metastasis subgroup was calculated on a per-patient basis (4/8, 50%). PR, partial response; RECIST, Response Evaluation Criteria in Solid Tumors; SD, stable disease.

During the initial 12 weeks of anlotinib therapy, all target lesions showed a reduction, with a mean reduction rate of 16% ± 7% (range, 5%-25%) in the longest diameter (Figure S1A). Five patients obtained further tumor shrinkage (1-4 mm), 3 had no measurable change, and 2 showed slight enlargement (2 mm and 4 mm, respectively) during levothyroxine withdrawal period. Following RAI therapy (weeks 13-24), additional lesion shrinkage of 5% ± 4% (range, 2%-12%) was observed in 5 patients, whereas the other 5 experienced mild tumor enlargement of 5% ± 3% (range, 2%-10%) (Figure S1B). Collectively, the sum of the longest diameters of target lesions demonstrated a mean reduction of 16% ± 7% (range, 7%-28%) from baseline after the first cycle of sequential therapy (Figure S1C).

Biochemical remission was reached in all patients (100%; 95% CI, 69%-100%; Figure S2). The median Tg_on_ level declined from 15 499 ng/mL (IQR, 694 to 21 216 ng/mL) to 256 ng/mL (IQR, 40-2320 ng/mL) (*P* < .001), with a mean δTg_on_% reduction of 88% ± 15% (95% CI, 78%-99%). The median biochemical TTR was 1.7 months (95% CI, 0.7-2.8 months; Figure 2A), substantially shorter than the median structural TTR of 15.7 months.

## Serious adverse events (SAEs)

No treatment-related serious adverse events, grade 4/5 treatment-related adverse events, or treatment-related deaths were reported.

## General toxicity profile

Treatment was generally well tolerated with no serious adverse events or grade 4/5 treatment-related toxicities observed; treatment-related adverse events are summarized in Tables 2 and 3.

**Table 2.** Treatment-emergent adverse events related to anlotinib (*n* = 10).

Adverse events^a^	Any grade no. (%)	Grade 1 no. (%)	Grade 2 no. (%)	Grade 3 no. (%)
**Hypertension**	6 (60)	1 (10)	2 (20)	3 (30)
**Hypercholesterolemia**	6 (60)	2 (20)	2 (20)	2 (20)
**Proteinuria**	7 (70)	5 (50)	2 (20)	0
**Hand-foot syndrome**	6 (60)	6 (60)	0	0
**Corrected QT prolongation**	2 (20)	2 (20)	0	0
**Elevated alanine aminotransferase**	2 (20)	1 (10)	1 (10)	0
**Oropharyngeal pain**	2 (20)	2 (20)	0	0
**Elevated low-density lipoprotein**	2 (20)	2 (20)	0	0
**Decreased platelet count**	1 (10)	1 (10)	0	0
**Leukocyturia**	1 (10)	1 (10)	0	0
**Anemia**	1 (10)	1 (100)	0	0

a Individual patient adverse events that have occurred multiple times are counted as a single event and the highest grade reported. No grade 4/5 events.

**Table 3.** Treatment-emergent adverse events related to ^131^I (*n* = 10).

Adverse events	Immediate (within 1 week) *n* (%)	Intermediate (2-4 weeks) *n* (%)	Long-term (5-12 weeks) *n* (%)
**Weakness**	6 (60)	2 (20)	0 (0)
**Neck pain**	5 (50)	0 (0)	0 (0)
**Neck swelling**	5 (50)	1 (10)	0 (0)
**Nausea**	4 (40)	0 (0)	0 (0)
**Stomachache**	2 (20)	0 (0)	0 (0)
**Acid reflux**	2 (20)	0 (0)	0 (0)
**Parotitis**	1 (10)	0 (0)	0 (0)
**Xerostomia**	0 (0)	0 (0)	1 (10)

## Discussion

This prospective, single-center, single-arm phase II trial did not complete its planned evaluable accrual. Of 24 enrolled patients, 14 (58%) were excluded because target lesions were non-RAI-avid on diagnostic ^1^^3^^1^I whole-body scan, leaving only 10 evaluable patients, below the prespecified 19. The trial was therefore underpowered to definitively assess the primary endpoint. Nevertheless, to our knowledge, this is the first trial to explore sequential TKI priming then RAI consolidation in distant metastatic RAI-avid DTC.

DTC accounts for approximately 95% of thyroid cancers, and distant metastases occur in about 10% of patients.^6^ RAI remains a cornerstone for RAI-avid disease, but its efficacy is limited, especially in macronodular lung metastases and bone lesions.^2^^,^^7^^,^^8^ Our previous work showed an ORR of approximately 35% for pulmonary metastases^8^ but only 3% for bone metastases with repeated high-dose RAI^2^. Angiogenesis is critical in thyroid cancer progression,^9^ and anlotinib, an oral multi-target TKI, has demonstrated efficacy in RAI-refractory DTC (median PFS 40.5 vs 8.4 months; ORR 59% vs 0%).^10^ We therefore hypothesized that anlotinib pretreatment could reduce tumor burden, favor subsequent RAI, and improve overall outcomes. The strategy also aimed to counteract thyrotropin surge-driven progression during levothyroxine withdrawal and to reduce cumulative toxicity through a planned 12-week anlotinib-free interval after RAI.

In the 10 evaluable patients, sequential anlotinib followed by 7.4 GBq RAI produced an ORR of 60% (6/10 PR) and a DCR of 100%; no complete responses occurred. Median structural TTR was 15.7 months, median biochemical TTR was 1.7 months, and biochemical remission occurred in all patients. At a median follow-up of 34.5 months, no progression or death occurred, and median PFS was not reached. In the subgroup of 8 patients with bone target lesions, ORR was 50%, which is notable because bone metastases historically respond poorly to RAI alone,^7^^,^^11^ 1 large study reported only 14/115 (12%) responses among younger patients with small bone metastases.^11^ Safety was manageable: grade 3 anlotinib-related AEs occurred in 30%, with no grade 4/5 events and RAI-related AEs were all grade 1. These findings are encouraging but hypothesis-generating.

The most important limitation is the absence of an RAI monotherapy control arm. Because all patients received anlotinib followed by RAI, the relative contribution of anlotinib priming versus RAI alone cannot be determined. During the first 12 weeks of anlotinib, all target lesions decreased by a mean of 16% ± 7% (range, 5%-25%), suggesting standalone activity in RAI-avid disease. After RAI, responses were heterogeneous: 5 patients had further shrinkage and 5 had mild enlargement. This pattern may reflect intrinsic RAI activity, radiation-induced inflammation, or assessment timing, and it does not establish causality for the sequential regimen. The observed ORR of 60% exceeds historical rates with RAI alone, but historical comparisons are confounded by differences in patient selection, tumor burden, and assessment schedules. Therefore, these data do not establish superiority over RAI monotherapy. Comparisons with concurrent TKI–RAI studies are also limited: apatinib and lenvatinib combinations have shown promising activity (ORR up to 67%),^12^^,^^13^ and Li et al. reported 55% ORR with concurrent anlotinib plus radioiodine but grade ≥3 anlotinib-related AEs in 50% and dose reductions in 50%.^14^ Our sequential approach yielded 60% ORR with grade 3 AEs in 30%, no grade ≥4 events, and 1 dose reduction, but cross-trial comparisons cannot establish superiority.

The trial also has limited statistical power. The original Fleming single-stage design assumed an ORR of 10% with RAI alone and targeted 50% with sequential therapy (one-sided α = 0.05; power = 80%; 19 evaluable patients). With only 10 evaluable patients, the prespecified power was not achieved. Using the original design assumptions, the actual power with 10 evaluable patients was approximately 83% only if the critical value was relaxed to ≥4 responses (one-sided α = 0.013); under the original α = 0.05, the study was substantially underpowered. Although 6/10 responses would be unlikely under a 10% ORR assumption (exact *P* ≈ .00015), the wide 95% CI (19%-81%) reflects substantial uncertainty. The small sample also precludes reliable subgroup analysis, including the bone metastasis subgroup. Additional limitations include the single-center design and follow-up insufficient to assess overall survival or late toxicity.

The high screen-failure rate at the RAI-avidity assessment stage is a key practical lesson. Future sequential TKI–RAI trials should incorporate pre-enrollment screening for RAI-avid target lesions to optimize patient selection and ensure adequate accrual of evaluable patients. In furture phase III trials, we would use a randomized, controlled, multicenter design with an RAI monotherapy control arm, prescreening for RAI avidity, pre-specified stratification by bone versus non-bone metastases, standardized response-assessment timing after RAI, and a larger sample size powered for PFS or overall survival. Correlative studies should be included to identify which patients benefit from TKI priming.

In conclusion, sequential anlotinib followed by RAI was associated with promising antitumor activity and manageable safety in RAI-avid distant metastatic DTC, including bone metastases. However, the small single-arm design and incomplete planned accrual preclude definitive conclusions. The current data do not establish superiority over RAI alone and should be considered hypothesis-generating. Randomized controlled trials with an RAI monotherapy arm are needed to validate this treatment concept.

## Supplementary Material

oyag426_Supplementary_Data

## Data Availability

Datasets generated in this trial are not publicly released owing to patient-privacy constraints. De-identified raw study data are available from the corresponding author upon reasonable written request.
