# Supplementary material for: Sequential therapy with anlotinib and radioiodine for distant metastatic differentiated thyroid cancer: a phase II clinical trial
Source: Oncologist. 2026 Oct 22;31(12):oyag426. doi: 10.1093/oncolo/oyag426 (PMC13750159; doi:10.1093/oncolo/oyag426)
Supplement: Supplementary file 1 [file oyag426_supplementary_data.zip › Supplement.doc]

**Appendix**

**Inclusion criteria**

1. Aged between 18 and 70 years at the time of signing informed consent, regardless of gender.
2. Confirmed history of locally advanced or metastatic differentiated thyroid carcinoma (DTC), documented by imaging or pathology.
3. Presence of at least one measurable lesion according to RECIST1.1 criteria.
4. ECOG performance status score of 0-1 and an estimated life expectancy of at least 24 weeks.
5. Baseline laboratory tests meeting the following criteria:
6. Hematology: Hemoglobin (HB) ≥90 g/L (without transfusion or erythropoietin (EPO) dependency within 14 days); Absolute neutrophil count (ANC) ≥1.5 × 10⁹/L; Platelet count (PLT) ≥80 × 10⁹/L.
7. Biochemistry: Total bilirubin (TBIL) <1.5 × upper limit of normal (ULN); Alanine aminotransferase (ALT) and aspartate aminotransferase (AST) <2.5 × ULN.
8. Renal Function: Serum creatinine (Cr) ≤1.5 × ULN or creatinine clearance ≥60 mL/min (calculated by Cockcroft-Gault formula).
9. Urinalysis: Urine protein < 2+.
10. Coagulation: Adequate coagulation function, with International Normalized Ratio (INR) and prothrombin time (PT) ≤1.5 × ULN.
11. Cardiac Function: Left ventricular ejection fraction (LVEF) ≥ lower limit of normal (50%) assessed by Doppler ultrasound.
12. Negative serum pregnancy test for women (e.g., women who have not undergone surgical sterilization or have been postmenopausal for less than 1 year) during screening. Both male and female subjects of childbearing potential must agree to use highly effective contraception from the time of signing informed consent until 6 months after study completion and must not donate sperm or ova.

**Exclusion Criteria**

1. History of other malignancies within the past 5 years, except for cured basal cell carcinoma of the skin or carcinoma in situ of the cervix.
2. Known contraindications to iodine-131 (¹³¹I) therapy, such as positive pregnancy test or lactation.
3. Previous treatment with tyrosine kinase inhibitors (TKIs) for anti-tumor therapy.
4. Anticipated lack of benefit from the next scheduled ¹³¹I therapy.
5. Uncontrolled hypertension despite treatment with two antihypertensive agents (systolic blood pressure >150 mmHg or diastolic blood pressure >100 mmHg).
6. Significant cardiovascular or cerebrovascular impairment, including but not limited to: congestive heart failure ≥ New York Heart Association (NYHA) class II; coronary artery disease; uncontrolled unstable angina; history of myocardial infarction or ischemic cardiomyopathy; history of stroke within 6 months prior to screening.
7. Sinus bradycardia > grade 1 (CTCAE v5.0), second-degree or higher atrioventricular block, or sinus arrest (except for patients with pacemakers).
8. Arrhythmia (including QTc interval ≥470 ms), or concurrent use of medications known to prolong the QTc interval, including antiarrhythmic therapy.
9. Active or uncontrolled severe infection (≥ CTCAE grade 2 infection).
10. Factors significantly affecting oral drug absorption (e.g., inability to swallow, chronic nausea/vomiting, gastrointestinal resection, chronic diarrhea, intestinal obstruction).
11. Bleeding tendency (e.g., active peptic ulcer) or requirement for anticoagulant therapy (e.g., warfarin, heparin, or analogues) or vitamin K antagonists.
12. Imaging evidence of tumor encasement of major blood vessels, or investigator judgment of high risk for life-threatening hemorrhage due to potential tumor invasion of major vessels during the study period.
13. Received external beam radiotherapy within 3 months prior to screening, or planning to receive systemic anti-tumor therapy (including cytotoxic therapy, signal transduction inhibitors, immunotherapy) concurrent with the study drug administration.
14. Planned concurrent use of traditional Chinese medicine for anti-tumor treatment.
15. Presence of non-healing wounds or fractures.
16. History of arterial/venous thrombotic events within 6 months prior to screening, such as cerebrovascular accident (including transient ischemic attack), deep vein thrombosis, or pulmonary embolism.
17. History of drug abuse (psychoactive substances) without ability to abstain, or history of psychiatric disorders.
18. Any other condition that, in the investigator's judgment, may jeopardize the subject, preclude compliance with study requirements, or otherwise make the subject unsuitable for study participation.

**Withdrawal Criteria**

1. Subject withdraws informed consent.
2. Development of concurrent illness significantly affecting clinical evaluation.
3. Receipt of non-study treatments that interfere with the study or use of prohibited medications specified in the protocol.
4. Any situation where the investigator deems it necessary for the subject to withdraw from the study.

**Study Termination Criteria**

1. Pregnancy.
2. Protocol violation rendering the subject unsuitable for further treatment (as determined by the investigator).
3. Disease progression or intolerable toxicity.
4. Any other situation deemed necessary for termination.
